# Intra-Ocular Lenses for Cataract Patients

**Published:** 1990-06

**Authors:** Neil L. Dallas

**Affiliations:** Consultant Ophthalmologist, Bristol Eye Hospital


### Intra-ocular Lenses for Cataract Patients

**Neil L. Dallas, TD, FRCS**
Consultant Ophthalmologist, Bristol Eye Hospital

1989 was the 40th anniversary of intra-ocular lens implantation. Harold Ridley performed the first implant for cataract surgery in November 1949 at St. Thomas' Hospital. The idea developed on the following lines: It was known that trauma from plastic imbedded in the eye was inert, a situation seen mainly in air pilots who had suffered injuries from shattered windscreens. But the seed was sown by a medical student who was watching his first cataract operation, and enquired whether a plastic lens could be re-inserted after the chrystalline one had been removed. It required a touch of genius to develop this, and Harold Ridley, with the co-operation of Rayner Optical, manufactured such a lens.

The visual results were dramatic. Up until then, cataract patients had been faced with thick magnifying lenses with distorted peripheral vision, the alternative being an uncomfortable contact lens, at that time in their infancy.

The operation, however, suffered complications, and although the new procedure was welcomed by many, it went through a period of bitter controversy which continued until at least the mid seventies. Ophthalmologists are a conservative group and many considered implant surgery as too dangerous to the patient's eye. For at least 10 years, therefore, Harold Ridley made few converts, and none of these were to be found amongst his colleagues at Moorfields. He very nearly abandoned the whole procedure. Let us see why.

The original IOL implant was a spherical lens, 9 mm. in diameter, placed behind the iris where the chrystalline lens had been removed. It was heavy and sometimes dislocated, or by pressure, caused glaucoma. Later, some corneas became cloudy as a result of operative damage (the Perspex CQ or polymethylmethacrylate is known to be hydrophobic and, therefore, a potential danger to the corneal endothelium). Later, an anterior chamber lens was designed and perfected over many years by Peter Choyce in Southend. This IOL was optically sound and did not dislocate. It did, however, continue to cause some corneal decompensation. The number of implant surgeons remained few and far between, most of them in the U.K. or the Netherlands.

In the 1960s, Cornelius Binkhorst produced a pre-pupillary lens with loops on either side of the iris to prevent dislocation. This operation was regarded as safer and by his scrupulous attention to detail, Binkhorst persuaded many colleagues to adopt the technique. Complications remained somewhat higher than in a plain cataract extraction, and by now more suitable contact lenses were available, including the extended wear soft ones.

Further changes occurred in the late 1970s. Micro-surgery was introduced, a visco-elastic substance named Healon could protect the surfaces of the plastic lens, and Kelman in the U.S. designed a phako-emulsification machine which could dissolve and wash-out the cataract through a small incision. The next generation of intra-ocular lenses, however, were again in the posterior chamber, but smaller and more physiologically acceptable to the eye. The posterior lens capsule had, of course, to be left intact to support the IOL. Only in 1978, therefore, did the numbers of implantations rise dramatically, principally because it was regarded as a safe and acceptable procedure in the U.S. Even in the U.K., the birthplace of the IOL, less than 10% of surgeons were prepared to implant eyes after cataract removal. I am glad to say that Bristol did better than that. C. A. Brown and Philip Jardine were experienced implant surgeons before I arrived in 1964; other colleagues were "occasional" implanters.

What is the state of intra-ocular lenses now? Over 100,000 IOLs are implanted each year in this country and well over 1,000,000 in the U.S. Nearly all eyes with cataract are suitable for IOLs and receive them. The operation has few complications and is one of the most successful operations in the world. This is just as well as, with an aging population, more and more cataract operations are carried out each year.

What of the early protagonists? Harold Ridley was made an FRS in 1988, and only the second ophthalmologist to be so honoured.

